# Combination of stem cell and gene therapy ameliorates symptoms in Huntington’s disease mice

**DOI:** 10.1038/s41536-019-0066-7

**Published:** 2019-03-26

**Authors:** In Ki Cho, Carissa Emerson Hunter, Sarah Ye, Alvince Learnz Pongos, Anthony Wing Sang Chan

**Affiliations:** 1 Department of Human Genetics, Emory University School of Medicine, Atlanta, GA USA; 2 Division of Neuropharmacology and Neurologic Diseases, Yerkes National Primate Research Center, Atlanta, GA USA

## Abstract

Huntington’s disease (HD) is a dominantly inherited monogenetic disorder characterized by motor and cognitive dysfunction due to neurodegeneration. The disease is caused by the polyglutamine (polyQ) expansion at the 5′ terminal of the exon 1 of the huntingtin (*HTT*) gene, *IT15*, which results in the accumulation of mutant HTT (mHTT) aggregates in neurons and cell death. The monogenetic cause and the loss of specific neural cell population make HD a suitable candidate for stem cell and gene therapy. In this study, we demonstrate the efficacy of the combination of stem cell and gene therapy in a transgenic HD mouse model (N171-82Q; HD mice) using rhesus monkey (*Macaca mulatta*) neural progenitor cells (NPCs). We have established monkey NPC cell lines from induced pluripotent stem cells (iPSCs) that can differentiate into GABAergic neurons in vitro as well as in mouse brains without tumor formation. Wild-type monkey NPCs (WT-NPCs), NPCs derived from a transgenic HD monkey (HD-NPCs), and genetically modified HD-NPCs with reduced mHTT levels by stable expression of small-hairpin RNA (HD-shHD-NPCs), were grafted into the striatum of WT and HD mice. Mice that received HD-shHD-NPC grafts showed a significant increase in lifespan compared to the sham injection group and HD mice. Both WT-NPC and HD-shHD-NPC grafts in HD mice showed significant improvement in motor functions assessed by rotarod and grip strength. Also, immunohistochemistry demonstrated the integration and differentiation. Our results suggest the combination of stem cell and gene therapy as a viable therapeutic option for HD treatment.

## Introduction

Huntington’s disease (HD) is a monogenic hereditary neurodegenerative disease characterized by progressive brain atrophy in the striatum, cortex, and other brain regions associated with cognitive, behavioral, and motor impairment.^1–5^ The causative factor of HD is the mutation in exon 1 of the huntingtin (*HTT*) gene resulting in the expansion of the polyglutamine (polyQ) residue at the N-terminus of the HTT protein.^1,6,7^ The onset and severity of the disease are governed by the size of the polyQ tract.^8^ The accumulation of oligomeric mutant HTT (mHTT) and the formation of nuclear inclusions are hallmark neuropathologies of the disease.^1,9,10^ However, the role of mHTT in HD pathogenesis remains unclear. Multiple proteolytic cleavage sites create unique splicing patterns in the HTT protein and produce a variety of N-terminal fragments.^1,6,7^ Moreover, the expanded polyQ tract creates aberrant splicing of the HTT protein that results in the formation of small oligomeric fragments.^1,9,11^ These oligomeric fragments fold, form aggregates, accumulate in cells, and disrupt cellular functions.^1,9,10^ Although there is a great advancement in the understanding of HD pathogenesis and the development of drugs and therapeutics, HD remains incurable and the search for effective treatments continues.

Recent advancement in cellular reprogramming technology provides a unique opportunity to derive induced pluripotent stem cells (iPSCs) from a patient’s own cells, making it an ideal cell source for personal stem cell replacement therapy with minimal or no immunological rejection. Similar to embryonic stem cells (ESCs), iPSCs are pluripotent and are capable of differentiating into many cell types of all lineages. However, for patients with inherited genetic mutations, such as expanded polyQ in HD, genetic correction prior to cell therapy is an inevitable step. Therapeutic effects of single-strand RNAs, mismatch-containing RNAs, antisense oligonucleotide (ASO), small hairpin RNA (shRNA), zinc finger nuclease (ZFN), and Clustered Regularly Interspaced Short Palindromic Repeats (CRISPR)-Cas9 targeting *mHTT* gene specifically or non-specifically have been reported.^12–22^ One of the major concerns of genome editing approach by ZFN or CRISPR/Cas9 is the non-specific targeting of the *mHTT* allele and irreversible editing of the genome, especially to the normal *HTT* allele. Although ablation of HTT in the adult mouse has no deleterious effect,^23^ long-term effects from HTT ablation and off-target effects from genome editing have yet to be determined. In a more recent study, ablation of *Htt* in adult mouse showed significant motor and behavioral decline.^24^ Unlike gene editing, gene silencing, such as shRNA and mHTT lowering therapy such as ASO, is a more validated treatment option for HD. Recent clinical trials of IONIS-HTTRx (ASO) and WVE-120101 (ASO) show promise as HD therapy.^25^ However, limitations in delivery method and biodistribution of the ASO, RNAi, and other therapeutic reagents in the brain remain major obstacles for clinical translation.

Transgenic HD monkey model was first reported in 2008 and longitudinal studies show progressive cognitive and motor impairment, progressive reduction in striatal volume and degeneration, decreased N-acetylaspartate (NAA), and progressive changes in whole brain white matter.^26–28^ These conditions recapitulate those observed in human HD patients that makes the HD monkey model a potential preclinical large animal model for assessing the efficacy of new therapeutics and treatments. Our team generated stable neural progenitor cell (NPC) cell lines from both WT and HD monkey iPSCs^29^ that are capable of differentiating into neurons in vitro.^22,30,31^ We further demonstrated intrastriatal NPC grafts in severe combined immunodeficiency (SCID) mice were capable of differentiating into neurons without tumor formation.^22^

In this study, we evaluated the efficacy of using a combination of stem cell therapy and gene therapy by grafting NPCs derived from WT and HD monkey, and HD-NPC expressing shRNA against *HTT* (HD-shHD-NPCs) in HD mice. We demonstrated that HD-shHD-NPC grafts significantly extended the lifespan of HD mice compared to a sham injection group. Also, both HD-shHD-NPC and WT-NPC groups showed significant improvement in motor function of HD mice compared to HD-NPC grafted and sham injection groups. Furthermore, we showed that grafted cells were capable of differentiating to MAP2, GABA, and GFAP expressing cells, which are markers for neurons and astrocytes. Our study demonstrates the combination of stem cell and gene therapy could be an effective treatment option for HD.

## Results

### Confirmation of HTT expression suppression by shHD

**Fig. 1:**
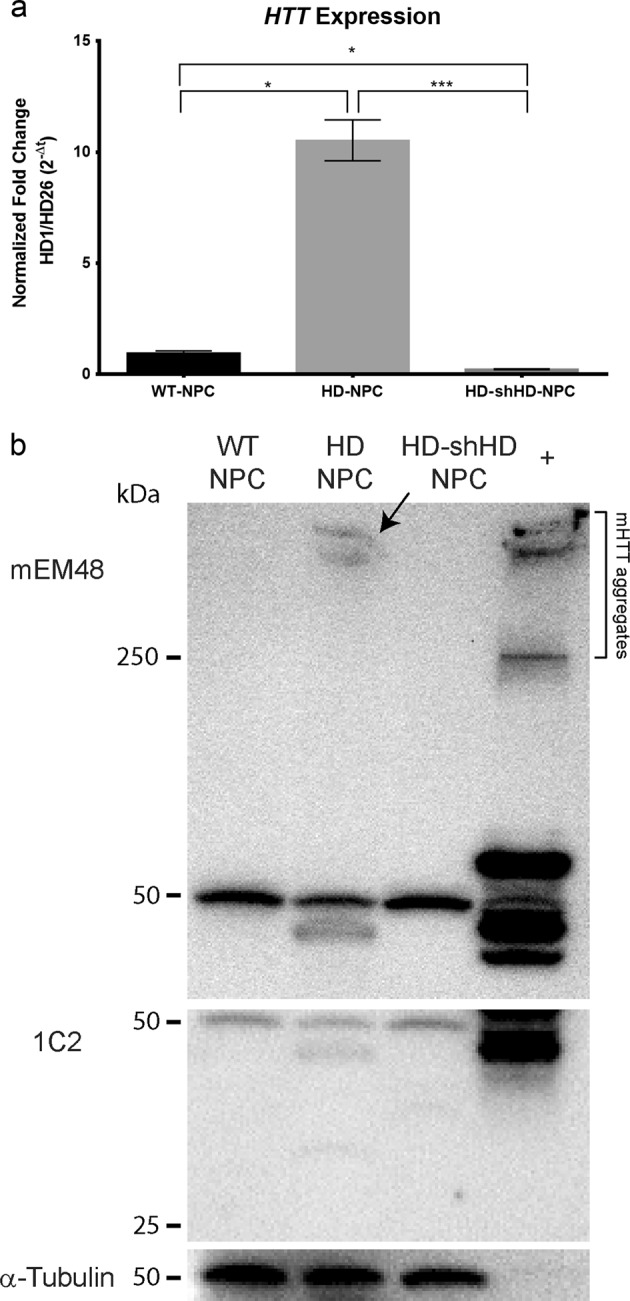
Suppression of HTT by expressing shHD. **a** qRT-PCR analysis of *HTT* exon 1 expression of HD-shHD-NPC showing significant suppression of *HTT* in HD-shHD-NPC compared to both HD-NPC (*P* < 0.0001) and WT-NPC (*P* = 0.003). Results shown from three biological replicates. Data is represented as mean ± SEM (*P < 0.05, ***P < 0.0001; ANOVA). **b** Western blot analysis showing increased oligomeric mHTT aggregates in stacking gel (mEM48) and soluble form (1C2) in HD-NPC while expression of shHD in HD-NPC decreased both aggregate accumulation in the stacking gel and soluble form of mHTT. Positive control used in this study was 293FT cells overexpressing expanded poly-Q (84Q) under the control of ubiquitin promoter

In our previous publication, we generated and extensively characterized NPCs that were used in this study including *HTT* gene expression and mHTT protein aggregates.^22^ In order to confirm the phenotype, HD-shHD-NPC cells were subjected to Zeocin selection (100 µg/mL), and the suppression of *HTT* expression was confirmed by using qRT-PCR before the surgery (Fig. 1a). HD-shHD-NPC showed significant reduction of HTT expression compared to both WT-NPC (*P* = 0.003) and HD-NPC (*P* < 0.0001) (Fig. 1a). Western blot analysis of WT-NPC, HD-NPC, and HD-shHD-NPC showed increased accumulation of oligomeric mHTT aggregates (mEM48) and soluble form of mHTT (1C2) in HD-NPC (Fig. 1b). The expression of shHD in HD-NPC decreased both aggregate accumulations in the stacking gel and soluble form of mHTT (Fig. 1b).

### Impact of HD-shHD-NPC grafts on rotarod and grip strength in HD mice

**Fig. 2:**
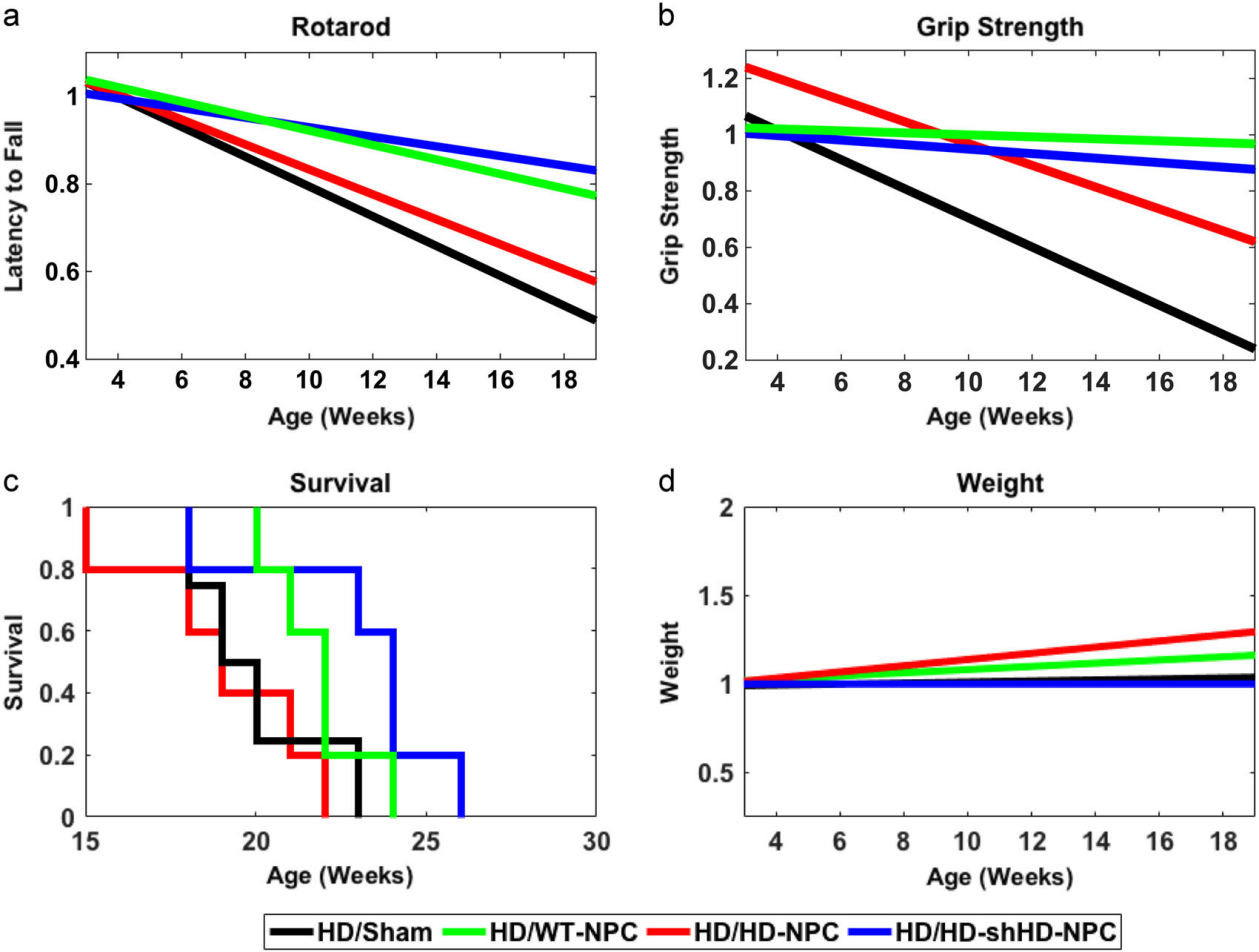
Longitudinal behavior assessments of HD mice grafted with NPCs. **a** Linear regression analysis of the rotarod data showed significant improvement in rotarod performance of the HD mice receiving WT-NPC (green) (*P* = 0.0468) and HD-shHD-NPC (blue) (*P* = 0.0026) compared to sham injection group (black). **b** Linear regression analysis of the grip strength data showed significant improvement of the grip strength in HD mice receiving WT-NPC (green) (*P* = 0.0014) and HD-shHD-NPC (blue) (*P* = 0.0077) compared to sham injection group (black). No statistical significance was found between HD-NPC and sham injection group (*P* = 0.7103). **c** Kaplan–Meier graph of HD mice grafted with NPCs. A significant increase in lifespan was observed when HD-shHD-NPC group was compared to the sham injection group ($\tilde x = 24.0$ weeks and $\tilde x = 19.0$ weeks) (*P* = 0.02). **d** Linear regression analysis of the weight did not show significant difference among the groups. HD/NT (*n* = 7), HD/Sham (*n* = 5), HD/WT-NPC (*n* = 5), HD/HD-NPC (*n* = 5), and HD/shHD-NPC (*n* = 5)

**Fig. 3:**
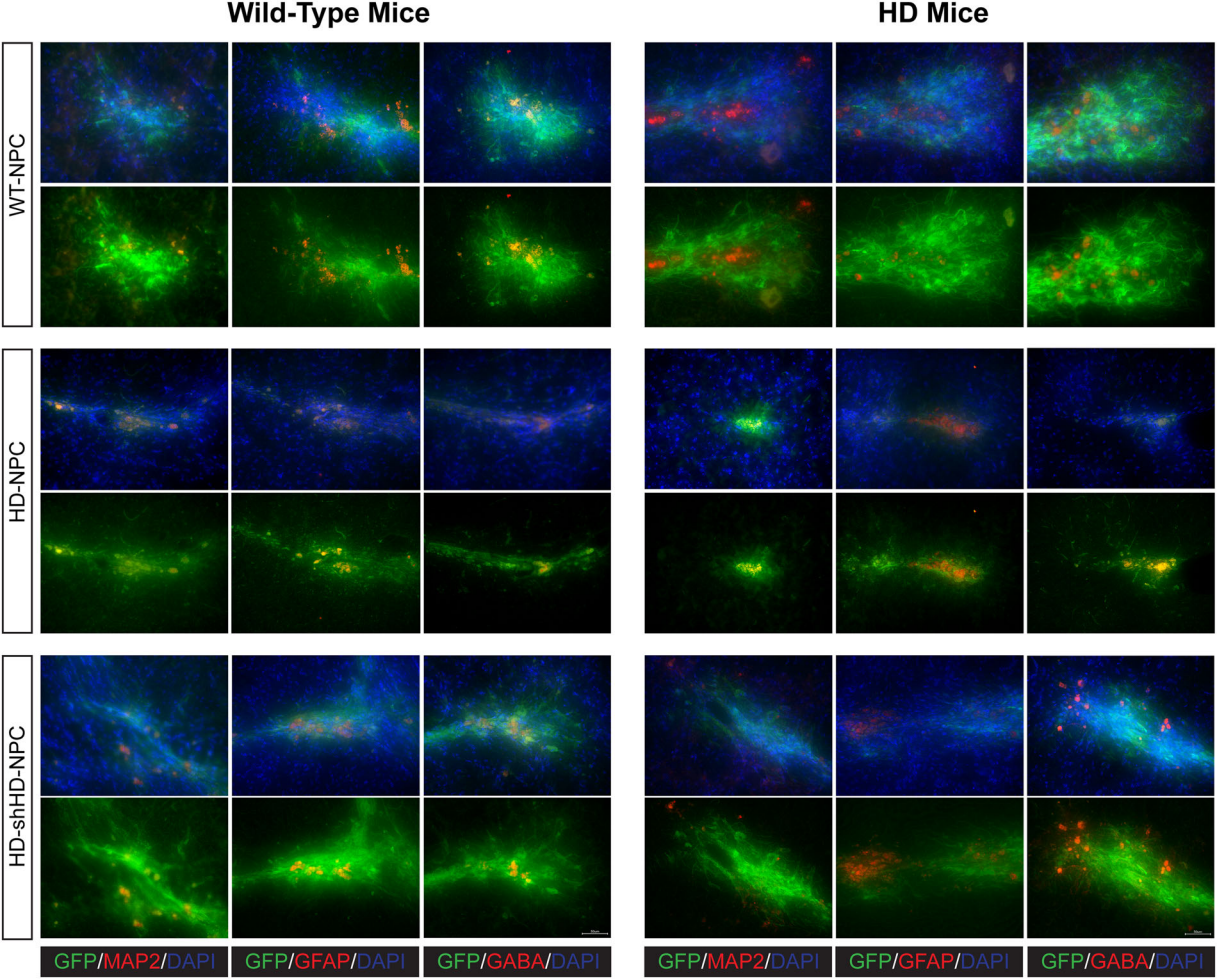
Immunohistochemistry reveals the differentiation of NPC grafts in mouse brains. The top panels show mouse brains with WT-NPCs grafts. The middle panels show mouse brains grafted with HD-NPCs. The bottom panels show mouse brains grafted with HD-shHD-NPCs. Wild-type mouse brains are shown on the left, and the HD mouse brains are shown on the right. More WT-NPCs survived, distributed, and differentiated into neural cells than compared to both HD-NPC and HD-shHD-NPCs. All three cell lines were able to differentiate into MAP2, GFAP, and GABA positive cells in vivo. All pictures were taken at 40×. The scale bar represents 50 µm

**Fig. 4:**
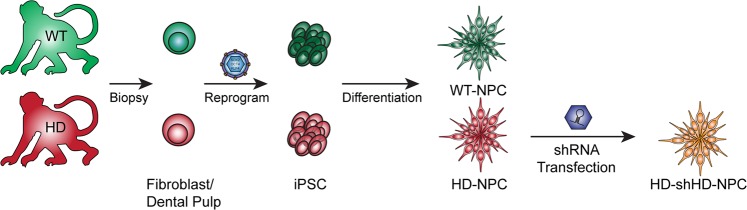
Derivation of cell lines from WT and HD monkeys. NPC cell lines were derived from monkey iPSCs via the reprogramming of the somatic cells. HD-NPCs were transfected with shRNA against *HTT* transcript and the resulted cell-line was named HD-shHD-NPC

A total of ten treatment groups with the number of mice used in each treatment group: WT/NT (*n* = 5), WT/Sham (*n* = 6), WT/WT (*n* = 6), WT/HD-NPC (*n* = 6), WT/shHD-HD-NPC (*n* = 6), HD/NT (*n* = 7), HD/Sham (*n* = 5), HD/WT-NPC (*n* = 5), HD/HD-NPC (*n* = 5), and HD/shHD-HD-NPC (*n* = 5), where first acronym denotes the genotype of mouse and second acronym denotes the treatment. In order to adjust for individual differences among different groups, all data for all groups, both WT and HD, were normalized to initial data point (Supplementary Figures 1, 2). All raw data is provided in Supplementary Figures (Figures 3, 4). Among all WT groups, no statistical significance was observed (Supplementary Figure 1). As expected, HD/WT-NPC and HD/HD-shHD-NPC showed similar performance in rotarod (*P* = 0.3479) and grip strength (*P* = 0.6563) (Table 1, Fig. 2a, b). Both HD/WT-NPC and HD/HD-shHD-NPC displayed slower behavioral performance deterioration than both HD/HD-NPC and HD/Sham grafts (Fig. 2a, b). HD/WT-NPC demonstrated better motor functions than that of HD/Sham (rotarod: *P* = 0.0468; grip strength: *P* = 0.0014) (Table 1, Fig. 2a, b). Notably HD/HD-shHD-NPC had similar improvement trajectory to HD/WT-NPC over HD/Sham in rotarod (*P* = 0.0026) and over HD/Sham in grip strength (*P* = 0.0077) (Table 1, Fig. 2a, b). However, no significant differences between HD/HD-shHD-NPC versus HD/HD-NPC and HD/WT-NPC versus HD/HD-NPC in grip strength and rotarod analysis were observed (Table 1). Additionally, no differences in rotarod nor grip strength progression were seen between HD/HD-NPC and HD/Sham (*P* = 0.7106 and *P* = 0.5356, respectively) (Table 1). Also, no significant differences in the weight of the HD mouse groups were observed (Fig. 2d).

**Table 1:** Pairwise comparison using Fisher’s protected LSD analysis of all HD mice

	Treatment 1	Treatment 2	95% Confidence intervals		*P*-value
			Lower	Upper	
Rotarod	Sham	WT-NPC	−0.0347	−0.0003	0.0468*
		HD-shHD-NPC	−0.0375	−0.0087	0.0026*
		HD-NPC	−0.0352	0.0242	0.7103
	HD-NPC	WT-NPC	−0.0183	0.0423	0.4297
		HD-shHD-NPC	−0.0123	0.0474	0.2422
	HD-shHD-NPC	WT-NPC	−0.0176	0.0064	0.3479
Grip strength	Sham	WT-NPC	−0.0762	−0.0201	0.0014*
		HD-shHD-NPC	−0.0750	−0.0124	0.0077*
		HD-NPC	−0.0550	0.0290	0.5356
	HD-NPC	WT-NPC	−0.0042	0.0745	0.0785
		HD-shHD-NPC	−0.0129	0.0743	0.1629
	HD-shHD-NPC	WT-NPC	−0.0158	0.0247	0.6563

Rotarod and grip strength tests of HD mice with different treatments were compared using Fisher’s protected LSD test implemented post-hoc on linear regressions to identify differences. For rotarod, HD mice that received intrastriatal WT-NPC injection showed significant improvement compared to the sham injection group (*P* = 0.0468), and mice that received intrastriatal HD-shHD-NPC showed considerable improvement (*P* = 0.0026). For grip strength, HD mice that received intrastriatal WT-NPC and HD-shHD-NPC injection showed significant improvement compared to the sham injection group (*P* = 0.0014, *P* = 0.0077, respectively) (* denotes *P* < 0.05)

### Overall impact of NPC grafts in HD mice

One-way ANOVA in HD mice with identical treatment groups to WT mice, resulted in significance from omnibus *F*-test for rotarod (*F*(4,119) = 4.83, *P* = 0.00119) and grip strength (*F*(4,108) = 7.64, *P* = 1.86e−5). For pairwise comparison of HD/Sham, HD/WT-NPC, HD/HD-NPC, and HD/HD-shHD-NPC trends, Fisher’s protected least significance difference (LSD) test was implemented post-hoc on linear regressions to identify differences in performance between treatment groups (Table 1, Supplementary Figure 2). One-way ANOVA analysis on WT mice behavioral assessments resulted in no significance of the omnibus *F* test for rotarod (*F*(4,158) = 0.93, *P* = 0.4503) and grip strength (*F*(4,157) = 0.56, *P* = 0.6938) (Supplementary Figure 1). Linear analysis on data from WT mice treatment groups revealed similar slopes within 95% confidence intervals of one another, highlighting the expected similar performances of all treatments in WT mice. Statistical analysis on WT mice behavioral assessments resulted in no significance of the omnibus *F* test for rotarod (*F*(4,112) = 0.68, *P* = 0.61) and grip strength (*F*(4,116) = 1.18, *P* = 0.32) (Supplementary Figure 1). Sham injection group was used as the control group for all comparisons because these mice received the same surgical procedures as well as pre- and post-surgical care.

### Impact of NPC grafts on lifespan of HD mice

NPC grafts did not affect the lifespan of WT mice. However, significant changes were observed when different HD groups were compared (Supplementary Figure 5a,b,c). HD-shHD-NPC grafts in HD mice significantly increased the lifespan ($\tilde x = 24.0$ weeks) compared to sham injection group ($\tilde x = 19.0$ weeks) (*P* = 0.02) (Fig. 2c). Although grafting of WT-NPCs showed a slight increase in the lifespan of the HD mice ($\tilde x = 22.0$ weeks) compared to the sham injection group, it was not statistically significant (*P* = 0.104). When both HD-shHD-NPC and WT-NPC groups were compared to no treatment group, both groups showed a significant increase in the lifespan (*P* = 0.0249 and *P* = 0.0482, respectively) (Supplementary Figure 5b).

### NPC grafts in HD mice

At the end of the study, brains were harvested, cryosectioned, and stained for neuronal markers. All three cell lines were tagged with GFP to distinguish grafted cells from endogenous brain cells. Two neural specific markers, MAP2 and GABA, and astrocyte-specific marker, GFAP, were used to determine the neuronal differentiation capability of the grafted cells. Immunostaining revealed GABA, MAP2, and GFAP positive cells co-labeled with GFP, which suggests successful neural differentiation of the NPC grafts (Fig. 3). However, WT-NPC grafts have better distribution and differentiation in both WT and HD mouse brains (Fig. 3). HD-NPC grafts showed the lower distribution compared to both WT-NPC and HD-shHD-NPC (Fig. 3). Compared to HD-NPC, HD-shHD-NPC grafts have better distribution and differentiation capability (Fig. 3). In this study (46 mice) as well as our previous study (24 SCID mice),^22^ no tumor formation was observed in mice that received NPC grafts.

## Discussion

The primary etiology of HD in humans is striatal neurodegeneration^32–35^ and striatal cell replacement therapy has been considered as a possible therapy for treating HD. Recent developments in stem cell technology, especially iPSCs,^36^ paved the way to derive and produce an unlimited source of autologous cells that can be used in regenerative medicine. Also, as a monogenic autosomal dominant disease, HD is a suitable candidate for gene therapy. The expression of mHTT causes dysregulation in global gene expression and cellular dysfunction,^37–39^ thus knock-out and knock-down of mHTT expression have been the focus of therapeutic development.^19,22,31,40–50^ Although autologous cells are the best source for cell replacement therapy, correction of the mutation is needed for long-term therapeutic effects. Therefore, by combining both stem cell replacement therapy and gene therapy, we sought to harness the advantages of both. Combining the two therapies offers many advantages: establishment of isogenic immune-compatible cells from patients, differentiation of target or precursor cell population, and genetic correction of mutants by knock-down or knock-out approaches by RNAi or CRISPR/Cas9. Here, we demonstrated that monkey NPC xenografts survived and ameliorated HD symptoms in HD mice (Fig. 4).

In order to capture the progressive nature of the neurodegeneration characteristics of HD, we conducted longitudinal behavioral assessments following cell transplantation. We also designed a statistical model to show the beneficial impact of stem cell transplants throughout the course of HD development. Grip strength and rotarod assessments underscore the positive impact of WT-NPC and HD-shHD-NPC grafts on the behavioral decline in HD mice. Importantly, NPC grafts with reduced expression of HTT/mHTT by overexpressing shHD (HD-shHD-NPC) (Fig. 1) showed significant improvement in rotarod study over sham group (Fig. 2a). Improvement in motor function and coordination supports the potential of NPC therapy to improve conditions of the impaired cell population.

Monkey NPC grafts survived, differentiated, and colonized to proximal areas of the injection site (Fig. 3). Specifically, WT-NPCs survived well in HD mice brains with extensive neurite development (Fig. 3). HD-NPCs remained locally at the graft site. In contrast, HD-shHD-NPCs differentiated and developed better neurite than HD-NPC in both WT and HD mice brains (Fig. 3). NPC grafts were capable of differentiating into different neuronal cell types, as revealed by the expression of MAP2, GFAP, and GABA that co-labeled with GFP (Fig. 3). Future work with additional neuronal markers and stereological studies will provide a quantitative assessment of this therapeutic efficacy. Also, due to the limitation of samples, we were not able to investigate gene expression in the brain with and without the NPC grafts. Differentially expressed genes among the treatment groups might provide insights into underlying mechanisms that caused poor neurite development of HD-NPCs in the brain and impact of WT-NPC graft in the HD brain. In future work, we will investigate differentially expressed genes among treatment groups.

HD mice that received HD-shHD-NPC lived about 5 weeks longer than the sham group, equivalent to 13.5 years in human years.^51^ Although WT-NPC slightly increased the lifespan of HD mice, about 3 weeks, it was not significant. This might be due to the small sample size or due to the more systemic nature of mHTT. There was no significant improvement in weight of HD mice received either WT-NPC and HD-shHD-NPC grafts (Fig. 2d). However, for humane treatment of the animals, all HD animals with movement disorders were supplied with soft-chow and hydrogels, which might have contributed to weight gain and longer lifespan. Our study was primarily focused on the striatal area, leading us to expect a relatively local rather than systemic impact. A recent study demonstrated that mice with conditional knock-out of HTT in postnatal age developed fatal acute pancreatitis, while adult HTT knock-out mice did not experience neuronal loss or a fatal phenotype.^23^ Using a similar approach, a recent publication reported motor and behavioral function decline, reduced lifespan, and extensive neuropathology, such as bilateral thalamic calcification in mice when Htt was eliminated in adult age (3–9 months), but they also reported that eliminating *Htt* in adult mouse (9 months) resulted in no obvious neuropathology in cortex and no impairment of medium spiny neuron survival.^24^ The authors speculate that genetic background differences (hemizygous versus homozygous normal *Htt* expression background) might have resulted in the different outcome.^24^ Dysregulation of *HTT* exerts a systemic rather than a brain-specific impact, highlighting that systemic approaches are needed when developing stem cell replacement therapy. Our previous study using mesenchymal stromal cell (MSC) showed that MSC grafts provide trophic support and facilitate local microenvironment in response to lesion such as degenerating neurons.^52^ Transplanting MSC recruited pre-existing neuronal cells to the graft sites, increased endogenous neurotrophic signaling, such as fibroblast growth factor (FGF2), ciliary neurotrophic factor (CNTF), vascular endothelial growth factor (VEGF), and nerve growth factor (NGF), and reduced the atrophy of the striatal volume in HD mice.^52^ The current study did not address such effects by NPC, and such an effect could not be eliminated. Whether the improvement is due to the neurotrophic signaling or cell replacement remains to be investigated.

Our study was designed based on published reports on NPC grafts.^22,53–55^ Different cell sources have been investigated in HD cell therapy, which include fetal neural cells (whole ganglionic eminence cell suspension), neural stem cells, NPCs, MSCs, and pluripotent stem cells (ESCs or iPSCs). So far, only fetal tissue has been used in HD clinical trial and with dismal outcome.^56^ Although most studies have demonstrated some degree of functional recovery with few studies reporting no positive outcomes at all after the transplantation (14 out of 42 studies),^56^ it is challenging or impossible to compare the outcomes from different studies due to difference in transplantation age, transplantation delivery method, functional outcome assessment period, heterogeneity in behavioral testing methods, and lack of in vivo monitoring of integration of the transplanted cells.^56,57^ Therefore, further research is needed to optimize surgical method, delivery method, frequency of the transplant, selection of optimal cell type(s), best number of cells to transplant, transplantation time/age, and most importantly is the biodistribution of the grafted cells. In recent studies, preconditioning with lithium chloride^58^ and lithium/valproic acid,^59^ co-transplantation,^60^ and overexpression of BDNF and NGF^61^ showed beneficial effect on survival and differentiation of stem cell grafts. Development in this area will improve the efficiency of stem cell therapy.

Gene therapy, such as RNAi,^22,31,40–44^ ZFN,^45^ and CRISPR/Cas9,^19,46,47^ has been investigated as a potential treatment option for HD. The inherent limitation of RNAi is partial suppression of gene expression, which might be advantageous in HD treatment because of the gain of function of mHTT and the loss of function of normal HTT potentially acting concomitantly in HD etiology.^56^ By knocking down the expression of *HTT*, which includes both *mHTT* and normal *HTT*, we showed the amelioration of the disease phenotype. Future studies on allele-specific shRNA or an inducible system to suppress *mHTT*, while sparing the expression of *HTT*, will help to improve the outcome. With ongoing clinical trials of IONIS-HTTRx and WVE-120101/2 (Phase 1/2a and Phase 1b/2a),^25^ RNAi technology is certainly a promising candidate in HD treatment.

To our knowledge, no other studies have reported the combination of RNAi and neural progenitor stem cell therapy in HD. We have demonstrated that the combination of gene and stem cell therapy can ameliorate HD symptoms in HD mice. Our results further underscore the potential of combining gene and personal cell replacement therapy in HD patients. Given the cognitive and psychiatric symptoms in HD, the evaluation of the efficacy of cell replacement therapy and gene therapy should include emotional, cognitive, and motor tests.^56^ To better evaluate therapeutic efficacy and safety of gene and stem cell therapy, non-human primate (NHP) models, such as the HD monkey, will provide a unique preclinical large animal model that could facilitate clinical translation of new therapeutic approaches. Recent success in stem cell therapies in NHPs^62–68^ and our recent report on developing HD model of NHP^26,27,69^ will facilitate the effort for clinical translation to benefit patients in need.

## Methods

### Animal care

This research and all procedures (e.g., husbandry, breeding, surgery, etc.) were reviewed and approved by the Institutional Animal Care and Use Committees (IACUC) of Emory University and in accordance with the Animal Welfare Act and the U.S. Department of Health and Human Services “Guide for the Care and Use of Laboratory Animals, 8th edition”. WT mice (B6C3F1/J) and HD mice (N171-82Q) were used in stereotaxic cell transplantation surgery (The Jackson Laboratory). The N171-82Q mice express an N-terminally truncated human huntingtin (HTT) cDNA that encodes 82 glutamines and first 171 amino acids transgene under the control of a mouse prion promoter. Mouse colonies were established with female wild-type mice bred with male HD mice. The genotypes were determined by PCR.

### Cell lines

NPCs were derived from WT monkey fibroblasts and HD monkey dental pulp stromal cells, which were previously reported.^22^ NHP NPCs used in our study express exon 1 of the human mHTT under the control of human polyubiquitin-C promoter (UBC).^6,7^ In brief, iPSCs were mechanically passaged onto Petri dishes, and cultured with mouse embryo fibroblast (MEF)-conditioned ES cells medium (R&D). Neurospheres were formed after culturing cells with derivation medium [DMEM/F12, 1× N2 (Invitrogen), 4 ng/mL bFGF (R&D), 2 mM L-glutamine, and 1× P/S (Invitrogen)] for 7 days. Neurospheres were transferred to poly-ornithine (P)/laminin (L) coated tissue culture plates [20 µg/mL poly-ornithine (Sigma) and 1 µg/cm^2^ laminin (Sigma)] and cultured with neural proliferation medium (NPM) containing Neurobasal medium (Life Technologies), 1× P/S (Invitrogen), 1× B27 (Life Technologies), 2 mM L-glutamine, 20 ng/mL bFGF (R&D), and 10 ng/mL mLIF (Chemicon). After 7–10 days, neural rosettes were manually passed onto a new P/L-coated culture dishes. NPCs were maintained and prepared by using published method.^22^ In brief, NPCs maintained in monolayer on P/L-coated plates in NPM. The medium was changed every 2 days and the cells were passaged once they reached 90–100% confluency. HD-NPCs expressing sh-RNA targeting the *HTT* transcript were established by lentiviral transfection of NPCs as previously described.^22^ All cell lines used in this study are available upon reasonable request.

### qRT-PCR

Total RNA was extracted using TRIzol® (Life Technologies) followed by DNA digestion using DNA-free^TM^ kit (Invitrogen). cDNA was synthesized using High-Capacity cDNA Reverse Transcription Kits (Applied Biosystems) using 500 ng of RNA samples. qRT-PCR was performed using either IQ^TM^ SYBR® Green (Applied Biosystems). CFX96 Real-Time Detection System (Bio-Rad) was used for the reaction. *HTT* gene expressions were normalized with ubiquitin-C (UBC) expression followed by exon 26 expression. The primer sequences are following: HD Exon 1 (forward 5′-GCGACCCTGGAAAAGCTGAT, reverse 5′-CTGCTGCTGCTGGAAGGACT), HD Exon 26 (forward 5′-ACCCTGCTCTCGTCAGCTTGG, reverse 5′-AGCAAGTTTCCGGCCAAAAT), and UBC (forward 5′-CCACTCTGCACTTGGTCCTG, reverse 5′- CCAGTTGGGAATGCAACAACTTTA).

### Western blot

Proteins were extracted using RIPA buffer from the cells, and the protein concentrations were quantified by Bio-Rad DC™ Protein Assay (Bio-Rad). Western blot was performed as described.^6^ Briefly, protein extracts were loaded and separated in 4% stacking and 9% resolving SDS-PAGE gel. Proteins were transferred to a PVDF membrane. Proteins were probed with primary antibodies mEM48 at 1:75 dilution (courtesy of Xiao-Jiang Li), 1C2 at 1:1000 dilution (MAB1574, Chemicon), α-Tubulin at 1:1000 dilution (T9026, Sigma), and peroxidase anti-mouse IgG at 1:10,000 (715-035-150, Jackson ImmunoResearch). All blots were derived from the same experiment were processed in parallel.

### Intrastriatal cell injection

**Fig. 5:**
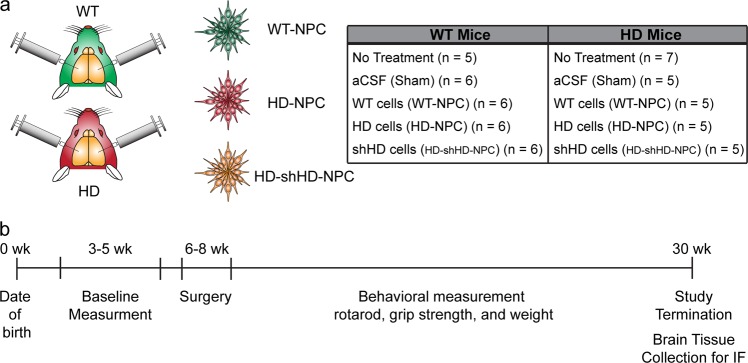
Summary of study design. **a** WT mice (B6C3F1/J) and HD mice (N171-82Q) were used in this study. A total of five treatment groups were designed in the study: no treatment (NT), artificial cerebrospinal fluid injected (aCSF or Sham), WT-NPC cells injected (WT-NPC), HD-NPC cells injected (HD-NPC), and HD-NPCs expressing shRNA against HD transcript injected (HD-shHD-NPC). **b** Behavioral assessments began with baseline measurements between 3 and 5 weeks of age. The surgery was conducted between 6 and 8 weeks of age, and all the behavioral assessments were repeated every 2 weeks subsequently until at least 18 weeks of age. Mice were euthanized between 19 and 32 weeks of age, depending on their conditions, and whole brains were collected for immunohistochemistry (IHC)

Cyclosporine A (Sandimmune®—Novartis) (10 mg/kg) and doxycycline (APP Pharmaceuticals) (2 mg/kg) injections were administered subcutaneously (SQ) daily for 3 consecutive days before surgery and 21 days post-surgery. On the day of surgery, mice were anesthetized using ketamine (Ketaset—Zoetis) (100 mg/kg) and dexmedetomidine (Dexdormitor—Zoetis) (0.5 mg/kg). Vital signs were monitored and recorded every 20 min during the surgery. WT-NPCs, HD-NPCs, and HD-shHD-NPCs were suspended in artificial cerebral spinal fluid (aCSF), and 2 µL of the cell suspensions (50,000 cells/µL) or 2 µL of aCSF were injected bilaterally into the striatum (anterior–posterior +0.74, medial–lateral ±1.7 relative to bregma, and dorsal/ventral = −3.8 from the surface of the skull) of 6–8 weeks old WT and HD mice using a rodent stereotaxic device (Lab Standard™—Stoelting) (Fig. 5). The injection was controlled by Micro 4^TM^ MicroSyringe Pump Controller (World Precision Instruments) at 500 nL per minute rate, and the needle was withdrawn at 5 min after completion of injection to minimize backflow of the cell suspension. Atipamezole hydrochloride (Anitsedan®—Zoetis) (1 mg/kg) was injected to reverse the sedative and analgesic effects, and meloxicam (Loxicom®—Norbrook) (2 mg/kg) injections were given before the surgery. Another dose of meloxicam (1 mg/kg) was given 24 h later and as needed. Daily observations by veterinary staff and lab personnel for the duration of the study were conducted to ensure proper recovery and health of animals. Additional analgesic was administered with any signs of pain or discomfort. For these experiments, the number of mice in each group were as follows (recipient mouse genotype/type of treatment): WT/NT (*n* = 5), WT/Sham (*n* = 6), WT/WT (*n* = 6), WT/HD-NPC (*n* = 6), WT/HD-shHD-NPC (*n* = 6), HD/NT (*n* = 7), HD/Sham (*n* = 5), HD/WT-NPC (*n* = 5), HD/HD-NPC (*n* = 5), and HD/HD-shHD-NPC (*n* = 5) (Fig. 5a).

### Behavior

To assess the impact of the grafts on fore- and hind-leg motor control, coordination, and the balance of mice, two behavioral assessments were conducted: rotarod and grip strength. All behavior assessments were done blind to treatment status. Assessments began between 3 and 5 weeks of age and then repeated every 2 weeks until 18 weeks of age. For rotarod, training began at around 4 weeks of age with three trials at a constant speed of 4 rpm and maximum trial length of 300 s. For these training periods, if mice fell off the rod they were placed back on for the duration of the 300 s. The rotarod test had a 20 s acceleration from 0 to 40 rpm. Three trials were conducted per session and a 2-min rest period was allotted between each trial. Latency to fall(s) was recorded for each trial by a sensor. For grip strength, mice were allowed to grip the grid of Grip Strength Test Device (Bioseb Inc.) with their fore-limbs. The tester gently pulled the tail in the opposite direction from the grid. The resulting muscular strength (*g*) was recorded. Grip strength assessments were performed by the same tester to minimize variation.

### Tissue preparation

Mice were euthanized between 19 and 32 weeks of age depending on their health condition. Transcardiac perfusion was performed using PBS with 4% paraformaldehyde (PFA). The brains were post-fixed, cryoprotected in 30% sucrose, and embedded in OCT compound. A serial coronal brain cryosection was performed at 30 µm thickness, mounted, and stored in −80 °C until further process.

### Immunohistochemistry (IHC)

Brain slices were stained for neuronal and astrocyte markers using the following primary antibodies: MAP2 (MAB3418—Chemicon) (1:500), GFAP (MAB360—Chemicon) (1:500), GABA (A2052—Sigma) (1:350), and GFP (G6539—Sigma) (1:500). The slices were incubated with the primary antibodies overnight at 4 °C. Alexa Fluor® 594 (Thermo) (1:1000), Alexa Fluor® 488 (Thermo) (1:1000), and Hoechst 33342 (Thermo) (1:1000) were applied the next day. Brain slices were then mounted and images were captured by using Keyence BZ-x700, with 40× objective and processed using BZ-X Analyzer (Keyence).

### Statistical analysis

For lifespan, log-rank (Mantel–Cox) test was used to test significance using GraphPad (GraphPad Software, Inc.). For all the other behavioral data sets, data were normalized to pre-surgical base-line. Using MATLAB’s “isoutlier” function, outliers’ more than 2.25 median absolute deviations from the median were removed. A critical value of *P* < 0.05 was considered significant for this study. One-way ANOVA analysis and linear regression analysis were used to determine whether there were significant differences among different treatment groups. Pairwise comparison using Fisher’s protected LSD test was implemented post-hoc on linear regression to identify differences in performance between graft treatments.

## Supplementary information

Supplementary Figures

## Data Availability

The raw behavior data are presented in Supplementary Data Set. The qRT-PCR and other behavior data generated during the current study are available from the corresponding author on reasonable request.

## Supplementary information

**Supplementary information** accompanies the paper on the *npj Regenerative Medicine* website (10.1038/s41536-019-0066-7).
