# Supplementary material for: Combination of stem cell and gene therapy ameliorates symptoms in Huntington’s disease mice
Source: NPJ Regen Med. 2019 Mar 26;4:7. doi: 10.1038/s41536-019-0066-7 (PMC6435637; doi:10.1038/s41536-019-0066-7)
Supplement: Supplementary file 1 — Supplementary Figures [file 41536_2019_66_MOESM1_ESM.pdf]

Supplementary Figure 1

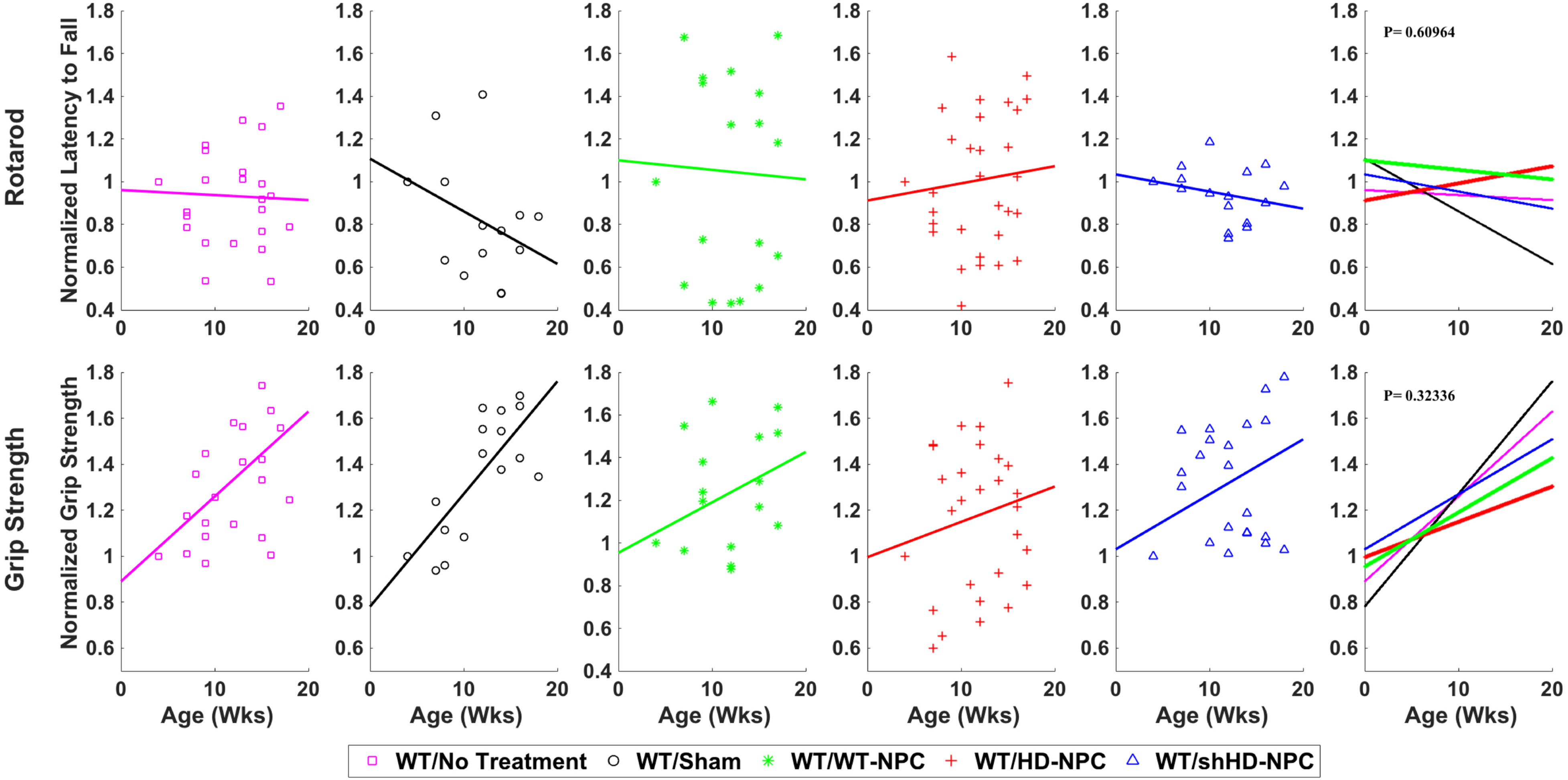

Supplementary Figure 1. Normalized Linear regression analysis of all WT mice.
WT/NT (n = 5), WT/Sham (n = 6), WT/WT (n = 6), WT/HD-NPC (n = 6),and WT/shHD-HD-NPC (n = 6).

Supplementary Figure 2

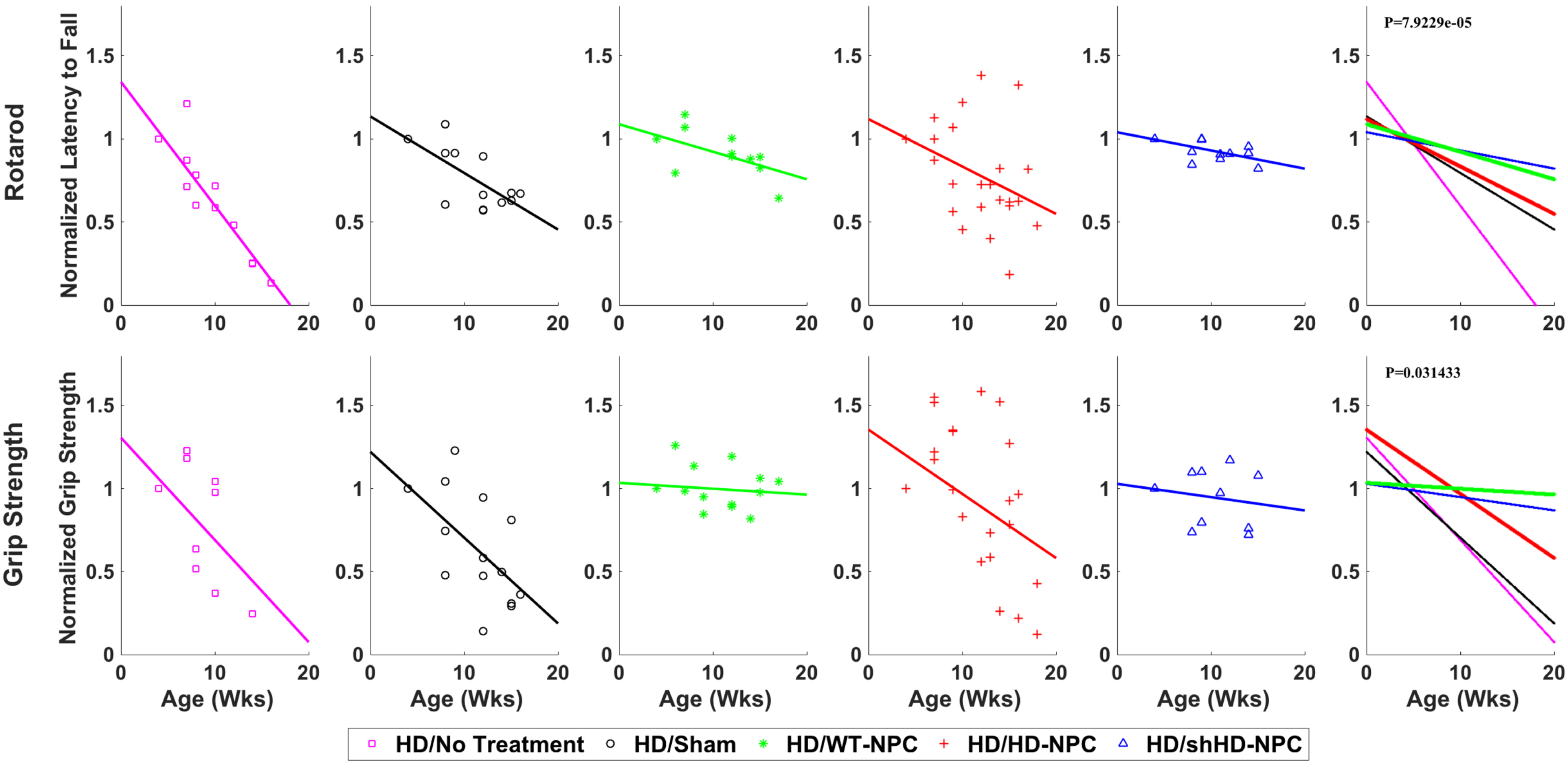

Supplementary Figure 2. Normalized Linear regression analysis of all HD mice.
HD/NT (n = 7), HD/Sham (n = 5), HD/WT-NPC (n = 5), HD/HD-NPC (n = 5), and HD/shHD-HD-NPC (n = 5).

Supplementary Figure 3

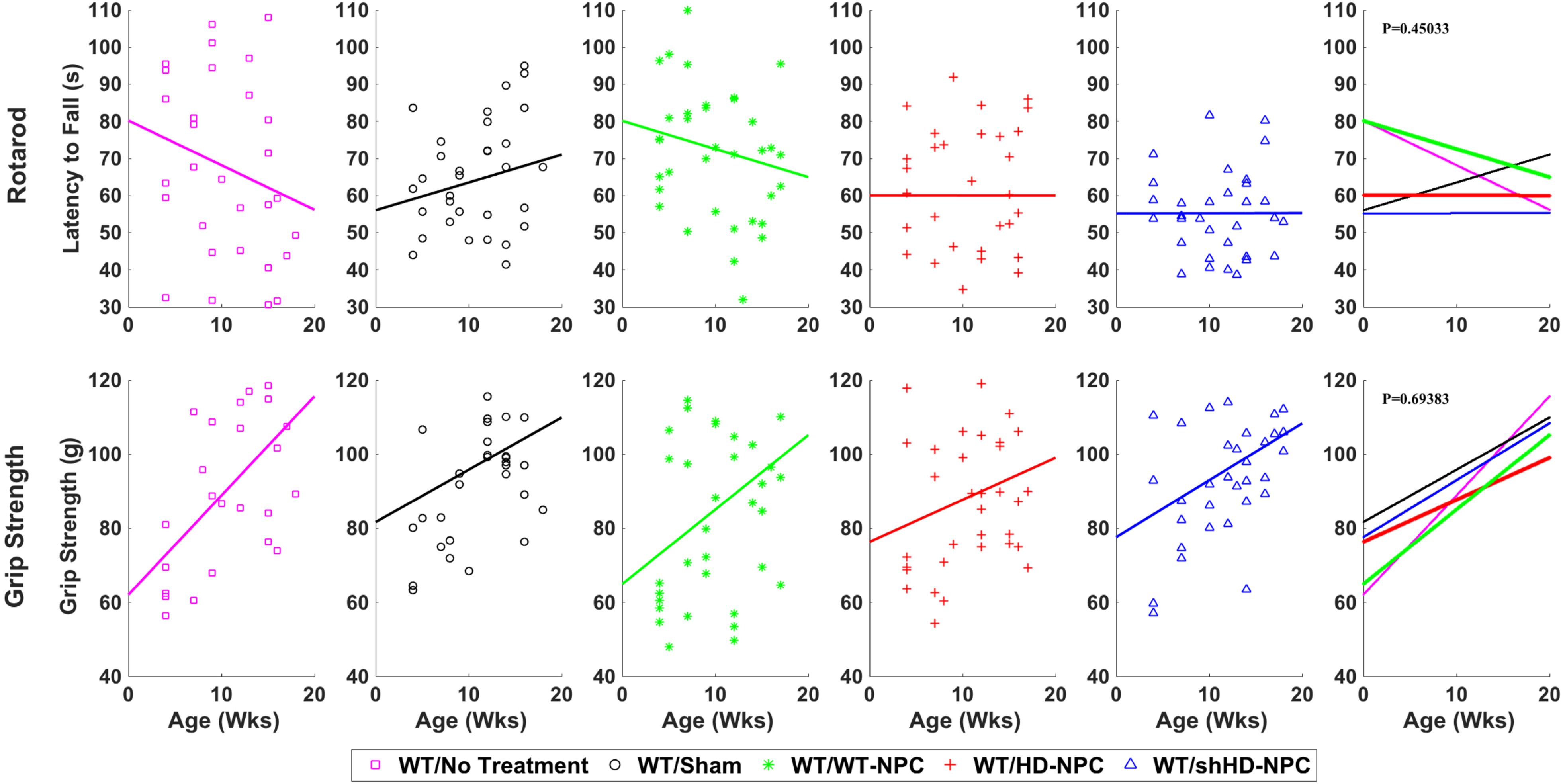

Supplementary Figure 3. Raw Data Linear regression analysis of all WT mice.
WT/NT (n = 5), WT/Sham (n = 6), WT/WT (n = 6), WT/HD-NPC (n = 6),and WT/shHD-HD-NPC (n = 6).

Supplementary Figure 4

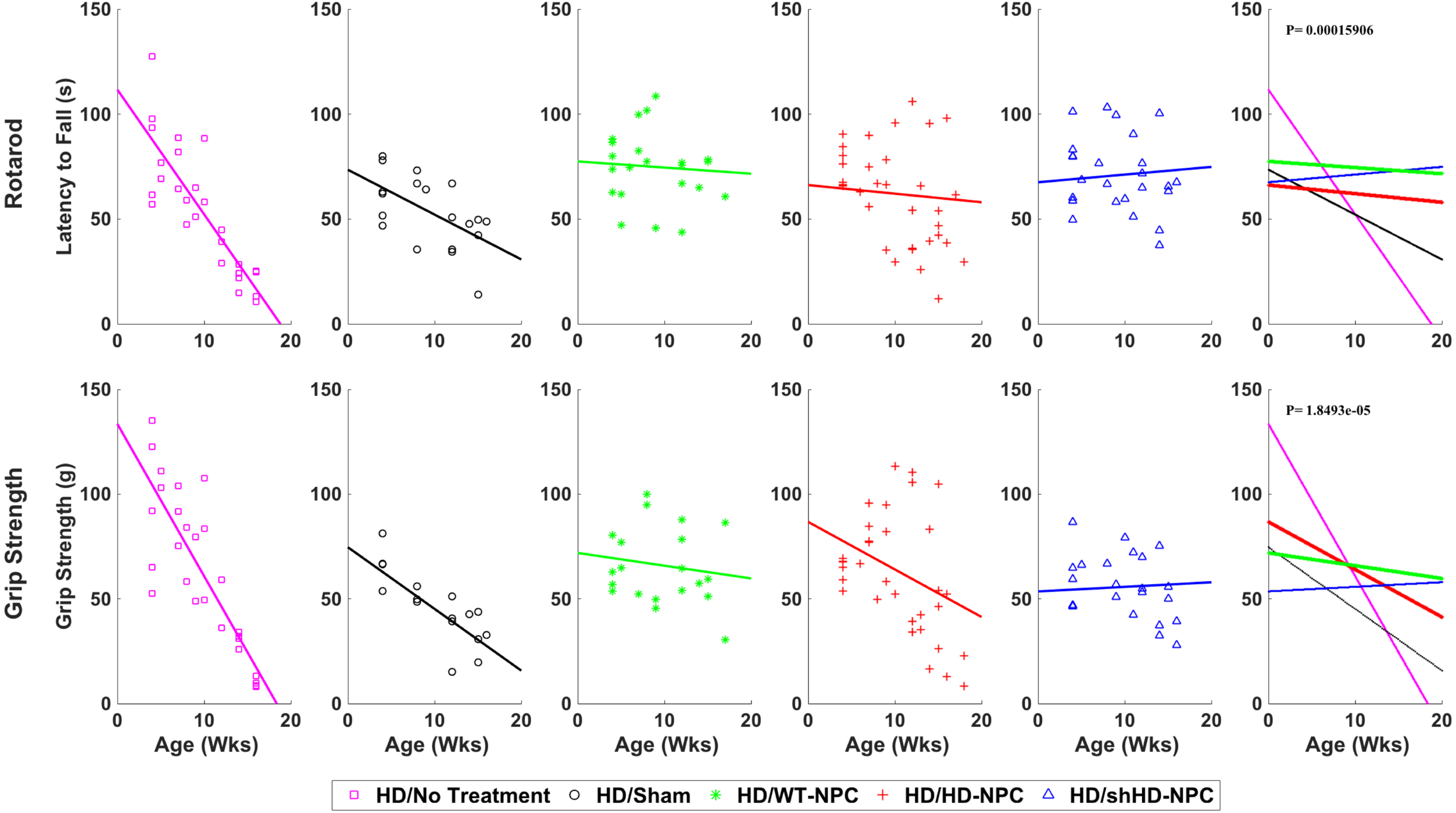

Supplementary Figure 4. Raw Data Linear regression analysis of all HD mice.
HD/NT (n = 7), HD/Sham (n = 5), HD/WT-NPC (n = 5), HD/HD-NPC (n = 5), and HD/shHD-HD-NPC (n = 5).

Supplementary Figure 5

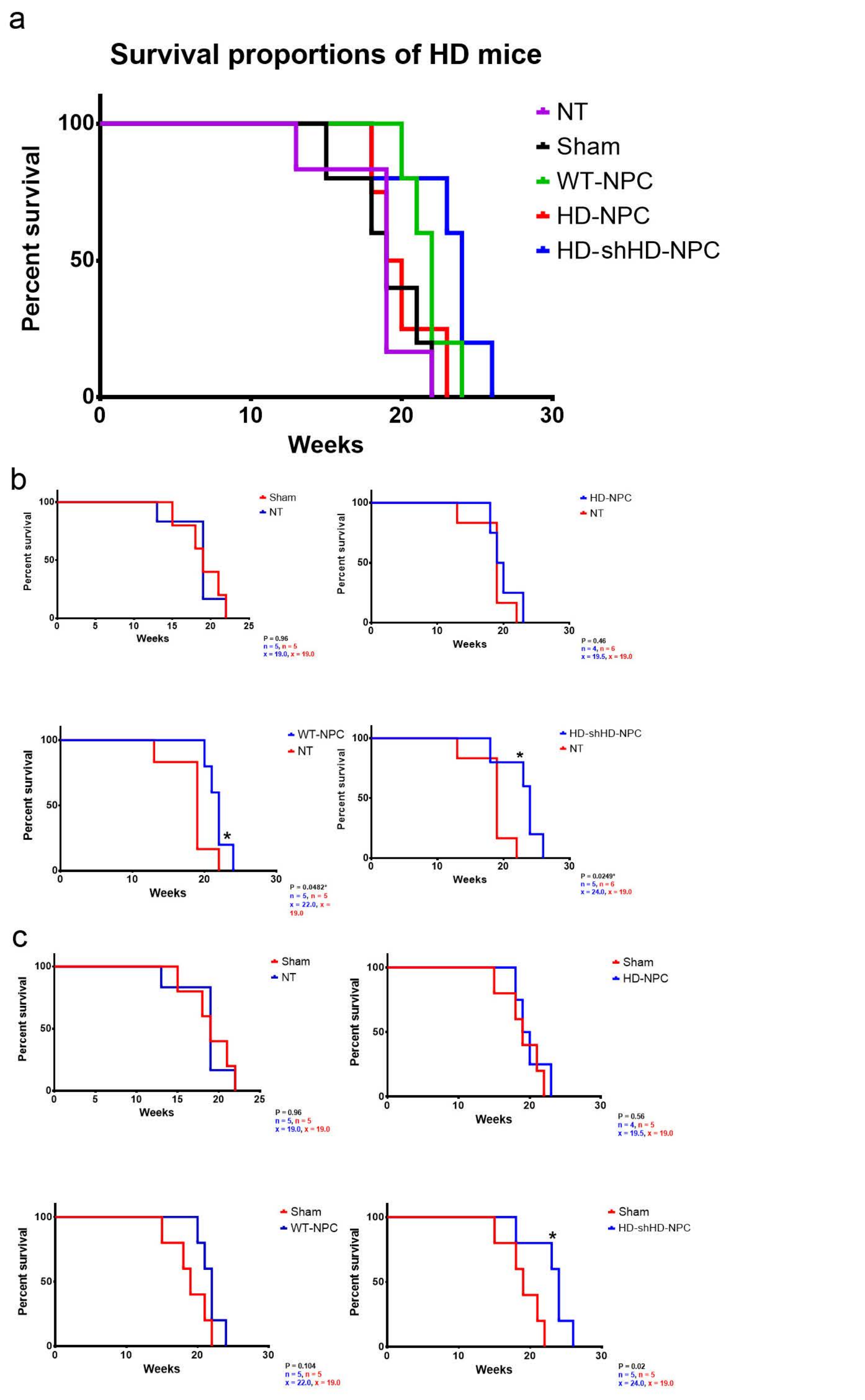

Supplementary Figure 5. Kaplan-Meier graph of HD mice.
(a) Survival graphs of all HD mice treatment groups.
(b) Statistical analysis compares each treatment group to no treatment (NT) group.
(c) Statistical analysis compares every treatment group to sham treatment group.
HD/NT (n = 7), HD/Sham (n = 5), HD/WT-NPC (n = 5), HD/HD-NPC (n = 5), and HD/shHD-NPC (n = 5).

Supplementary Figure 6

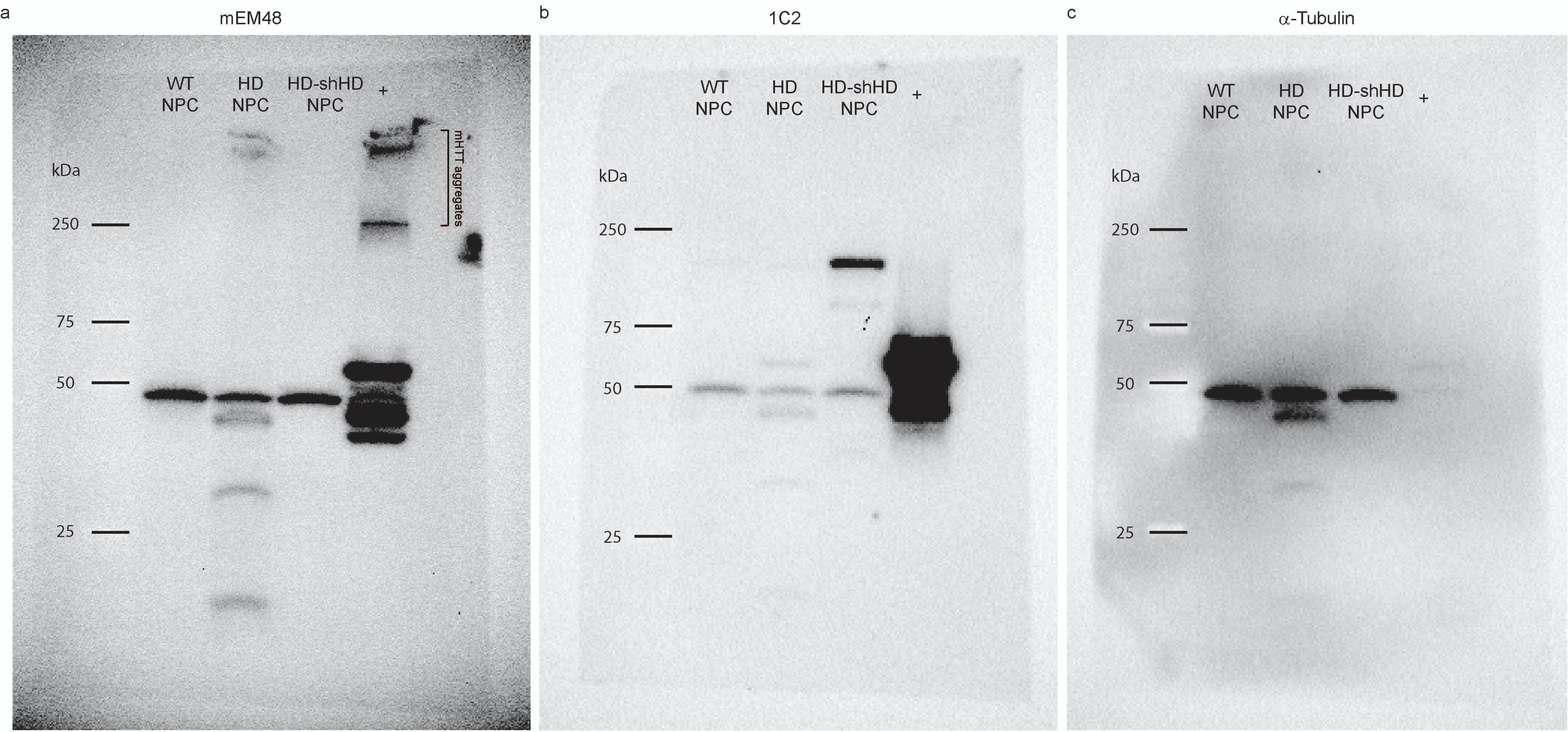

Supplementary Figure 6. Uncropped images of Western Blot. (a) Western blot of all samples using mEM48 antibody. (b) Western blot of all samples using 1C2 antibody. (c) Western blot of all samples using α-tubulin antibody.
